# Chronic Eosinophilic Pneumonia: A Diagnostic Challenge With Atypical Bronchoalveolar Lavage Findings and an Organizing Pneumonia Pattern

**DOI:** 10.1002/rcr2.70780

**Published:** 2026-09-30

**Authors:** Yang‐Tai Lin, Wei‐Fan Ou, Sha‐Yen Liang, Yu‐Hsuan Hu, Wan‐Ching Chen, Ming‐Cheng Chan

**Affiliations:** ^1^ Department of Chest Medicine Taichung Veterans General Hospital Taichung Taiwan; ^2^ Department of Radiology Taichung Veterans General Hospital Taichung Taiwan; ^3^ Department of Pathology and Laboratory Medicine Taichung Veterans General Hospital Taichung Taiwan; ^4^ Department of Critical Care Medicine Taichung Veterans General Hospital Taichung Taiwan

**Keywords:** bronchoalveolar lavage, chronic eosinophilic pneumonia, eosinophilic lung disease, organizing pneumonia, surgical lung biopsy

## Abstract

Chronic eosinophilic pneumonia (CEP) is a rare eosinophilic lung disease. We report a 61‐year‐old man with asthma who presented with progressive exertional dyspnoea for 2 months and worsening productive cough for 2 weeks. Peripheral blood eosinophilia was 15% (1314 cells/μL), and chest computed tomography showed bilateral peripheral, upper‐lobe‐predominant opacities. Bronchoalveolar lavage (BAL) revealed marked neutrophil predominance (85%) with only 6% eosinophils. Empirical antibiotics produced no clinical or radiographic improvement. Because clinical and radiological suspicion for CEP remained high despite discordant BAL findings, surgical lung biopsy was performed, demonstrating prominent eosinophilic infiltration with an organizing pneumonia pattern, supporting a diagnosis of CEP. The patient responded well to systemic corticosteroids. This case highlights the role of surgical lung biopsy in suspected CEP with atypical BAL findings and illustrates that organizing pneumonia may occur within the histopathological spectrum of CEP.

## Graphical abstract

A 61‐year‐old man with asthma, peripheral eosinophilia, and characteristic peripheral opacities was suspected of having chronic eosinophilic pneumonia (CEP), despite bronchoalveolar lavage showing marked neutrophil predominance and only 6% eosinophils. Surgical lung biopsy revealed prominent eosinophilic infiltration with an organizing pneumonia pattern, supporting the diagnosis of CEP. This case highlights that discordant BAL findings do not exclude CEP and that organizing pneumonia may occur within its histopathological spectrum.

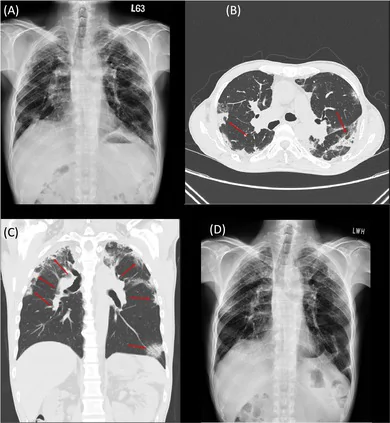

## 1 Introduction

Chronic eosinophilic pneumonia (CEP) is an eosinophilic lung disease characterized by prominent eosinophilic infiltration of the lung parenchyma. It typically affects middle‐aged adults, shows a female predominance, and is strongly associated with asthma and atopic disease, with up to two‐thirds of patients having a history of asthma. Clinically, CEP usually follows a subacute to chronic course, with cough, progressive dyspnoea, and constitutional symptoms developing over several weeks to months. Radiologically, bilateral peripheral‐predominant consolidations and/or ground‐glass opacities are characteristic, while marked eosinophilia in bronchoalveolar lavage (BAL) fluid or peripheral blood commonly provides evidence of eosinophilic inflammation [1, 2].

Although no universally accepted diagnostic criteria exist for CEP, diagnosis generally relies on a compatible subacute clinical course, characteristic peripheral‐predominant pulmonary infiltrates, evidence of blood or BAL eosinophilia, and exclusion of secondary causes of pulmonary eosinophilia. BAL eosinophilia is typically marked, often ≥ 40%, while peripheral blood eosinophilia of ≥ 1000 cells/μL is also commonly observed. In patients with typical clinical, radiological, and eosinophilic findings, lung biopsy is generally unnecessary and is usually reserved for cases with atypical or inconclusive features [3].

We describe an unusual case of CEP with neutrophil‐predominant BAL findings that initially suggested pneumonia. Surgical lung biopsy subsequently demonstrated prominent eosinophilic infiltration with an organizing pneumonia pattern.

## 2 Case Report

A 61‐year‐old male ex‐smoker presented with progressive exertional dyspnoea for 2 months and worsening productive cough over the preceding 2 weeks, accompanied by weight loss. He had a history of asthma maintained on inhaled fluticasone furoate/vilanterol, type 2 diabetes mellitus treated with empagliflozin/metformin, and hyperlipidaemia treated with simvastatin for over 10 years. He reported no recent travel abroad and no consumption of raw or undercooked food. Physical examination revealed bilateral fine inspiratory crackles without wheezing. There was no purpura, mechanic's hands, Gottron papules, Raynaud phenomenon, synovitis, or motor or sensory deficit suggestive of mononeuritis multiplex. Laboratory testing showed peripheral eosinophilia of 15% (1314 cells/μL). Chest radiography demonstrated bilateral peripheral patchy opacities (Figure 1A), while high‐resolution computed tomography revealed multifocal peripheral consolidations with upper‐lobe predominance and traction bronchiectasis (Figure 1B,C).

**FIGURE 1:**
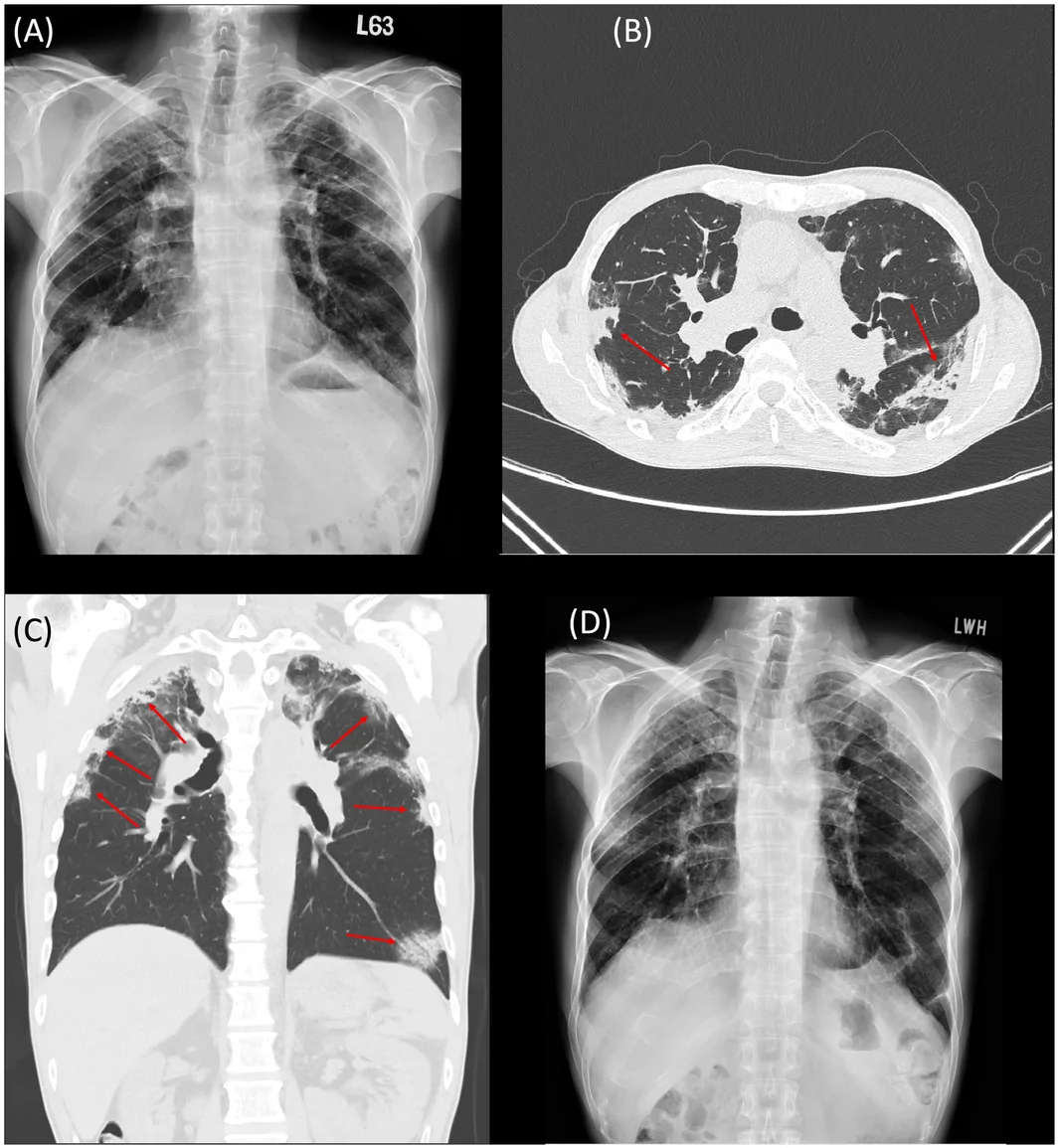
Chest radiograph and high‐resolution computed tomography. (A) Chest radiograph obtained at admission demonstrated peripheral opacities with upper‐lobe predominance. (B, C) High‐resolution computed tomography demonstrated bilateral confluent consolidations and ground‐glass opacities with an upper‐lobe and peripheral subpleural predilection (red arrows). (D) Chest radiograph obtained after approximately 1 month of corticosteroid treatment demonstrated resolution of the previously noted peripheral opacities.

CEP was initially suspected based on the subacute clinical course, characteristic radiological findings, and peripheral blood eosinophilia. However, bronchoalveolar lavage (BAL) showed marked neutrophil predominance (85%) with only 6% eosinophils. Autoimmune serological tests, including antinuclear antibody, proteinase 3‐antineutrophil cytoplasmic antibody, and myeloperoxidase‐antineutrophil cytoplasmic antibody, were negative, and the total IgE level was 289 kU/L. Urinalysis showed neither haematuria nor proteinuria, and renal function was normal. Given the BAL findings, an infectious process was initially favoured, and empirical amoxicillin/clavulanate was administered. However, his symptoms and radiographic abnormalities did not improve. Further BAL microbiological cultures were negative, no parasitic organisms were identified on cytological examination, and stool examination for parasites was also negative.

Video‐assisted thoracoscopic surgery wedge resection of the left lower lobe was subsequently performed. Histopathological examination demonstrated an organizing pneumonia pattern with active immature fibrosis and prominent eosinophilic inflammatory infiltrates involving the interstitial and peribronchiolar regions (Figure 2A,B), without evidence of vasculitis (Figure 2C). After integrating the clinical course, peripheral eosinophilia, radiological findings, histopathological features, and assessment for secondary causes, a diagnosis of CEP was made. Intravenous methylprednisolone was commenced at 40 mg daily, equivalent to prednisolone 1 mg/kg/day, then converted to oral methylprednisolone before discharge and gradually tapered over approximately 2 months. Symptoms and radiographic abnormalities improved progressively, and a chest radiograph obtained after approximately 1 month of corticosteroid treatment showed resolution of the previously noted bilateral peripheral opacities (Figure 1D).

**FIGURE 2:**
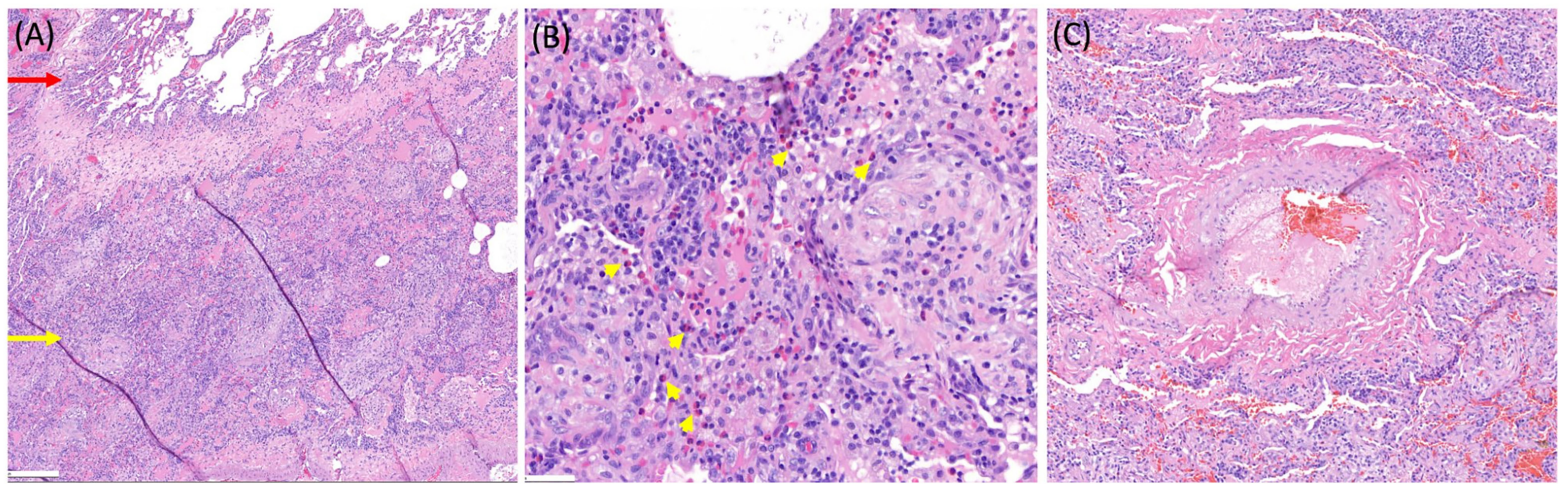
Pathology of wedge resection of left lower lobe. (A) Low‐power view (original magnification × 5) of haematoxylin and eosin (H&E) staining showed relatively preserved alveolar architecture (red arrow) and intra‐alveolar proliferation of fibroblastic foci with associated inflammatory cell infiltration (yellow arrow). (B) High‐power view (original magnification × 20) of H&E stain showed fibroblastic foci (Masson bodies) accompanied by an inflammatory infiltrate composed predominantly of eosinophils (yellow arrowheads), together with plasma cells and scattered lymphocytes. (C) A blood vessel shows an intact wall with no histological evidence of vasculitis.

## 3 Discussion

We present a case of CEP with atypical neutrophil‐predominant BAL findings and an organizing pneumonia pattern on surgical lung biopsy. Despite the atypical BAL profile, integration of the clinical, radiological, and histopathological findings supported the diagnosis.

In typical CEP, lung biopsy is generally not required when the clinical presentation, radiological pattern, and eosinophilic findings are concordant. In our patient, however, BAL demonstrated marked neutrophil predominance with only 6% eosinophils, despite peripheral blood eosinophilia and radiological features suggestive of CEP. Although BAL eosinophilia is usually prominent in CEP, considerable variability has been reported, with eosinophil proportions ranging from 12% to 95% in previous series [2]. Furthermore, BAL samples only a limited portion of the distal airways and alveolar compartment, and its cellular profile should be interpreted in conjunction with the clinical and radiological findings rather than used in isolation. A normal or atypical BAL differential does not exclude microscopic abnormalities in lung tissue [4]. Therefore, when BAL findings are discordant with the clinical and radiological features, lung biopsy may be considered in selected patients to establish the diagnosis.

CEP and cryptogenic organizing pneumonia (COP) share several clinical and radiological features, including subacute respiratory symptoms, pulmonary consolidation, and ground‐glass opacities, which may complicate their distinction. Histologically, however, organizing pneumonia (OP) represents a non‐specific pattern of lung injury and may occur in a variety of pulmonary disorders, including CEP. Although eosinophilic infiltration of the alveolar spaces and interstitium is a characteristic histopathological feature of CEP, foci of OP with intra‐alveolar fibroblastic plugs may also be present [5]. Therefore, the presence of an OP pattern does not necessarily indicate COP. In our patient, the combination of an OP pattern with prominent eosinophilic infiltration, together with compatible clinical, radiological, and peripheral blood findings, supported the diagnosis of CEP [1].

In conclusion, this case highlights the importance of integrating clinical, radiological, and BAL findings in the diagnosis of eosinophilic lung disease. BAL cellular analysis is supportive and should not be used alone to exclude CEP when other features remain suggestive. In selected patients with discordant findings, surgical lung biopsy may help establish the diagnosis. In addition, CEP may show eosinophilic infiltration accompanied by an organizing pneumonia pattern on histopathology.

## Author Contributions

**Yang‐Tai Lin:** conceptualization, investigation, writing – original draft, writing – review and editing. **Wei‐Fan Ou:** investigation, writing – original draft, writing – review and editing. **Sha‐Yen Liang:** validation. **Yu‐Hsuan Hu:** validation. **Wan‐Ching Chen:** validation. **Ming‐Cheng Chan:** conceptualization, supervision.

## Funding

The authors have nothing to report.

## Ethics Statement

The study was approved by the Institutional Review Board and Ethics Committees of Taichung Veterans General Hospital (approval number: CE251026C) and conducted in accordance with the Declaration of Helsinki.

## Consent

The authors declare that written informed consent was obtained for the publication of this manuscript and accompanying images and attest that the form used to obtain consent from the patient complies with the Journal requirements as outlined in the author guidelines.

## Conflicts of Interest

The authors declare no conflicts of interest.

## Data Availability

The data that support the findings of this study are available on request from the corresponding author. The data are not publicly available due to privacy or ethical restrictions.
